# Cortical asymmetry in Parkinson's disease: early susceptibility of the left hemisphere

**DOI:** 10.1002/brb3.573

**Published:** 2016-10-06

**Authors:** Daniel O. Claassen, Katherine E. McDonell, Manus Donahue, Shiv Rawal, Scott A. Wylie, Joseph S. Neimat, Hakmook Kang, Peter Hedera, David Zald, Bennett Landman, Benoit Dawant, Swati Rane

**Affiliations:** ^1^ Department of NeurologyVanderbilt UniversityNashvilleTNUSA; ^2^ Vanderbilt University Institute of Imaging ScienceNashvilleTNUSA; ^3^ Meharry Medical CollegeNashvilleTNUSA; ^4^ Department of NeurosurgeryUniversity of LouisvilleLouisvilleKYUSA; ^5^ Department of BiostatisticsVanderbilt UniversityNashvilleTNUSA; ^6^ Department of PsychologyVanderbilt UniversityNashvilleTNUSA; ^7^ Department of Electrical EngineeringVanderbilt UniversityNashvilleTNUSA

**Keywords:** cortex, left hemisphere, MRI, neurodegeneration, Parkinson's disease

## Abstract

**Background and Purpose:**

Clinically, Parkinson's disease (PD) presents with asymmetric motor symptoms. The left nigrostriatal system appears more susceptible to early degeneration than the right, and a left‐lateralized pattern of early neuropathological changes is also described in several neurodegenerative conditions, including Alzheimer's disease, frontotemporal dementia, and Huntington's disease. In this study, we evaluated hemispheric differences in estimated rates of atrophy in a large, well‐characterized cohort of PD patients.

**Methods:**

Our cohort included 205 PD patients who underwent clinical assessments and T1‐weighted brain MRI's. Patients were classified into Early (*n *= 109) and Late stage (*n *= 96) based on disease duration, defined as greater than or less than 10 years of motor symptoms. Cortical thickness was determined using FreeSurfer, and a bootstrapped linear regression model was used to estimate differences in rates of atrophy between Early and Late patients.

**Results:**

Our results show that patients classified as Early stage exhibit a greater estimated rate of cortical atrophy in left frontal regions, especially the left insula and olfactory sulcus. This pattern was replicated in left‐handed patients, and was not influenced by the degree of motor symptom asymmetry (i.e., left‐sided predominant motor symptoms). Patients classified as Late stage exhibited greater atrophy in the bilateral occipital, and right hemisphere‐predominant cortical areas.

**Conclusions:**

We show that cortical degeneration in PD differs between cerebral hemispheres, and findings suggest a pattern of early left, and late right hemisphere with posterior cortical atrophy. Further investigation is warranted to elucidate the underlying mechanisms of this asymmetry and pathologic implications.

## 1 Introduction

Over the course of Parkinson's disease (PD), degenerative changes progress throughout the neural networks subserved by the basal ganglia and cerebral cortex, resulting in cortical and subcortical atrophy. Advancing disease pathology, characterized by cerebral alpha‐synuclein deposition, is thought to contribute to these observations (Braak & Braak, 2000), and the rate and pattern of cortical changes appear to be a result of the duration of disease (Braak et al., 2003; Hawkes, Del Tredici, & Braak, 2010). Indeed, the location of cortical involvement depends on the stage of PD, most often tied to duration of disease or severity of clinical symptoms. Structural MRI studies suggest that frontal brain regions (insula, orbitofrontal, dorsolateral frontal, and middle temporal cortices) are susceptible to atrophy early in the course of disease, while more posterior regions (cuneus/precuneus and occipital cortex) tend to show changes in more advanced PD (Kempster, Gibb, Stern, & Lees, 1989; Wang et al., 2015).

In addition to the anterior/posterior distinction that emerges over the progression of PD, there is an important component of hemispheric lateralization over the course of the disease. Clinically, PD is well recognized as an inherently asymmetric disease, with a unilateral onset of motor symptoms. This clinical asymmetry is associated with more severe contralateral nigrostriatal degeneration (Kempster et al., 1989; Wang et al., 2015). Despite this well‐described localization, several studies suggest increased “left hemisphere susceptibility,” in that the left nigrostriatal pathway is more affected than the right (Prakash, Sitoh, Tan, & Au, 2012; Scherfler et al., 2012). Some suggest this may be an effect of handedness (Barrett, Wylie, Harrison, & Wooten, 2010; van der Hoorn, Burger, Leenders, & de Jong, 2011; Uitti, Baba, Whaley, Wszolek, & Putzke, 2005), but handedness does not account for this observation entirely. Given the apparent susceptibility of the left nigrostriatal network to degeneration, the nature of cortical changes in PD may also lateralize. Several studies observe PD‐related cortical changes in the left hemisphere (Bruck, 2004; Mak et al., 2014), and this may reflect the extent of disease progression (Jubault et al., 2011). Left hemisphere‐predominant asymmetry appears to be most evident early in the disease course, whereas increasing disease duration results in more diffuse and widespread brain atrophy. The left hemisphere‐predominant pattern of cortical atrophy is not unique to PD but is well‐described across the spectrum of neurodegenerative disorders, including Alzheimer's disease (Donix et al., 2013; Shi, Liu, Zhou, Yu, & Jiang, 2009; Thompson et al., 2003), frontotemporal dementia (Rohrer et al., 2012; Whitwell et al., 2013), and Huntington disease (Beste et al., 2009; Lambrecq et al., 2013; Muhlau et al., 2007).

In this study, we sought to further investigate hemispheric asymmetry of cortical atrophy over the course of disease in a large cohort of PD patients with varying disease duration. We hypothesized that asymmetric cortical atrophy occurs early in PD, mirroring the left greater than right nigrostriatal degeneration previously described. We further accounted for handedness and side of motor symptom severity to ascertain if asymmetric progression is explained by factors such as neurodevelopment or motor severity.

## 2 Materials and Methods

### 2.1 Patients

This cohort consisted of 205 consecutive patients who presented to Vanderbilt University between 2006 and 2013 and completed a clinical evaluation for consideration of Deep Brain Stimulation (DBS). All patients were diagnosed with idiopathic PD by a neurologist trained in Movement Disorders and met UK Brain Bank criteria for a diagnosis of PD (Hughes, Daniel, Blankson, & Lees, 1993), including documented motor improvement to dopamine therapy. Motor severity was defined using part III of the United Parkinson Disease Rating Scale (UPDRS) and was performed by a single examiner both in the withdrawn dopamine state (overnight withdrawal) and in the optimized medicated state (Fahn et al., 1987). In addition, patients and caregivers underwent detailed interview assessments defining the month and year of the first motor symptom. From this, disease duration was estimated based on the time from first symptom (or in rare cases when unclear, first diagnosis date) to current assessment. The Vanderbilt University Institutional Review Board approved the study, and all patients provided informed consent for the clinical protocol.

### 2.2 MRI acquisition

As part of the DBS imaging protocol, a sedated 3 Tesla T1‐weighted anatomical MRI (Philips Medical Systems, Best, The Netherlands) with body transmit and eight‐channel SENSE array‐coil reception was performed for placement of bone markers. Brain imaging was obtained using a 3D turbo field echo acquisition with a spatial resolution* *= 1 × 1 ×  1 mm^3^, slices = 170, TR/TE = 7.92/3.65 ms.

### 2.3 Cortical Thickness Measurement

A standard FreeSurfer pipeline (http://surfer.nmr.mgh.harvard.edu/, v5.1.0) was applied to all the T1‐weighted MRI images to intensity normalize, skull‐strip, and segment brain tissue (Fischl & Dale, 2000). The choice of imaging parameters closely matched the FreeSurfer recommended parameters. Han et al. (2006) describe that segmentation inaccuracies are minimal with FreeSurfer‐recommended imaging parameters, and do not affect study outcome; thus, reducing the need to verify segmentation accuracy for each subject. In order to ensure accuracy of segmentation for this analysis, we randomly selected and checked 15% of processed images. Each subject's data were registered to the standard FreeSurfer brain (fsaverage). The Destrieux atlas was used to identify cortical brain regions and evaluate cortical thickness per region (Destrieux, Fischl, Dale, & Halgren, 2010).

### 2.4 Regional and temporal variations in cortical thinning

To formally test our hypotheses that asymmetric cortical loss occurs earlier in disease and that different brain regions atrophy at different rates, we categorized patients into two populations based on disease duration. Disease duration was defined based on time since first motor symptoms, which was determined by the neurologist examining the patient through historical reference from the patient and caregiver as well as outside records, if available. PD patients with disease duration of ≤10 years were designated as early disease stage (Early), while patients with PD >10 years were designated as late disease stage (Late). This designation was guided by previous reports that indicate that patients in the second decade of disease have greater rates of cortical atrophy in widespread brain regions (Zarei et al., 2013) as well as symptoms of increasing disease severity, including cognitive impairment (Hobson & Meara, 2004; Reid, Hely, Morris, Loy, & Halliday, 2011; Verbaan et al., 2007) and gait instability (Factor et al., 2010; López, Ruiz, del Pozo, & Bernardos, 2010). The relation between cortical thickness and disease duration was estimated for each group separately, using bootstrapped linear regression.

### 2.5 Statistical approach

We first determined if cortical thickness differences distinguished Early from Late duration PD patients. Here, we computed a *p*‐value associated with the difference at each region, with 95% CI, using a two‐sample *t* test. The statistical significance of a group of *p*‐values in each hemisphere was assessed while controlling FDR at 0.1. Second, we estimated linear rates of cortical atrophy in the 75 brain regions of the Destrieux atlas in each group and then mapped these findings onto a 3‐Dimensional brain. Rates of atrophy were estimated as the slope of the linear regression. Bootstrapped differences between the slopes for Early and Late groups (Early–Late) were also estimated in each region. Lower and upper 95% confidence intervals for the bootstrapped differences between duration groups were estimated to determine significance. Regions with negative lower and upper 95% confidence intervals were considered to show significantly greater (*p *< .05) early stage atrophy, while regions with positive lower and upper 95% confidence intervals were considered to show significantly greater (*p *< .05) late‐stage atrophy.

## 3 Results

### 3.1 Patient population

Of the 205 patients in this cohort, the mean age was 62.2 (±8.9) years, mean disease duration was 10.1 (±4.5) years, and the majority (*n *= 138) were male. Table 1 outlines detailed clinical and demographic information. An priori cutoff of 10 years, which was just below the mean disease duration for the cohort, resulted in 109 patients classified as Early and 96, classified as Late. The mean age at assessment did not differ between those classified as Early or Late in disease (*t* test, *p *= .14), but the age of motor symptom onset was significantly lower in those classified in the Late group (*p *< .001). UPDRS III motor (Off) score did not differ significantly between groups (*p *= .2); yet, the Late disease duration group had a slightly lower On medication UPDRS III severity, and thus greater improvement on medication (i.e., UPDRS improvement on dopamine medication was significantly greater in the Late group) (*p *= .002).

**Table 1:** Demographic and clinical characterization of the PD cohort

	Total group (*n *= 205)	Early stage (*n *= 109)	Late stage (*n *= 96)
Gender (M:F)	138:67	71:38	67:29
Age‐assessment (years)	62.2 (8.9)	61.3 (9.3)	63.2 (8.4)
Range	30.1–82.7	30.1–82.7	40.8–82.1
Age‐Motor Onset (years)	52.1 (9.2)	54.4 (9.2)	49.3 (8.5)^a^
Range	26.0–78.0	26.0–78.0	30.0–72.0
Disease Duration (years)	10.1 (4.5)	6.9 (2.0)	13.8 (3.8)
Range	2.2–27.1	2.2–9.9	10.1–27.1
UPDRS III‐ Off	41.1 (12.6)	40.0 (12.4)	42.3 (12.8)
Range	12–84	12–84	16–78
UPDRS III‐ On	18.9 (10.0)	19.9 (11.0)	17.8 (8.8)
Range	0–45	0–45	3–44
UPDRS Difference	22.1 (10.2)	20.1 (9.5)	24.5 (10.6)^b^
Range	4–58	4–50	6–58
Handedness (R:L:NR^+^)	171:21:14	92:10:7	79:11:8

+: Not recorded.

Data is presented as mean (standard deviation).

a Significant differences between Early‐ and Late‐stage group (*t* test, *p *< .001).

b *p *= .0019.

### 3.2 Cortical thickness differences between Early and Late groups

Regions with significant differences in cortical thickness in Early versus Late groups are summarized in Table 2. Difference values and *p*‐values are presented after correction for multiple comparisons with FDR <0.1. The longer disease duration PD group displayed significant reductions in cortical thickness: (a) in bilateral occipital lobe/ posterior cortical regions, and (b) in a right hemisphere frontal–parietal predominant pattern. These right‐lateralized areas included the anterior cingulate, frontal pole, inferior supramarginal gyrus, and the subparietal sulcus. Overall, these results emphasize that despite a similar age at assessment, patients in the second decade of motor symptoms have reduced cortical thickness in key regions previously identified as susceptible to late‐stage PD. We then investigated whether the rates of cortical atrophy were different in these regions early versus late in the disease course.

**Table 2:** Difference values and *p*‐values for regions that differed significantly after correction for multiple comparisons using FDR <0.1

Region	Difference (EL = 0 – EL = 1) (95% CI)	*p*‐value
Right Hemisphere		
Mid. Frontal sulcus	0.0614 (0.0109, 0.1119)	.01752
Inferior precentral sulcus	0.0594 (0.0105, 0.1082)	.01748
Anterior Cingulate gyrus + sulcus	0.0763 (0.0139, 0.1388)	.01690
Precuneus gyrus	0.0641 (0.0116, 0.1166)	.01690
Mid. Occipital gyrus	0.0912 (0.0209, 0.1615)	.01134
Sup. Occipital gyrus	0.0872 (0.0246, 0.1497)	.00653
Lateral. Occipito‐temporal gyrus	0.0819 (0.024, 0.1398)	.00580
Lat Sulcus, Anterior/ Vertical	0.1122 (0.0338, 0.1907)	.00528
Subparietal sulcus	0.0916 (0.0276, 0.1556)	.00524
Inf. Frontal sulcus	0.0661 (0.0211, 0.111)	.00416
Frontal pole gyrus + sulcus	0.0771 (0.0256, 0.1287)	.00353
Sup. Occipital sulcus	0.0769 (0.0272, 0.1267)	.00260
Lat sulcus, Anterior/ Horizontal	0.1208 (0.0482, 0.1935)	.00122
Lat. Occipito‐temporal sulcus	0.1057 (0.0475, 0.1639)	.00043
Left Hemisphere		
Ant. Circ. Insula sulcus	0.1084 (0.0376, 0.1791)	.00286
Mid. Occipital gyrus	0.1016 (0.0358, 0.1674)	.00266
Sup occipital sulcus	0.076 (0.0278, 0.1241)	.00214
Lat sulcus, Anterior/ Horizontal	0.1243 (0.0486, 0.2001)	.00142
Sup. Occipital gyrus	0.1003 (0.0412, 0.1593)	.00097

### 3.3 Cortical rates of atrophy: early and late in disease

**Figure 1:**
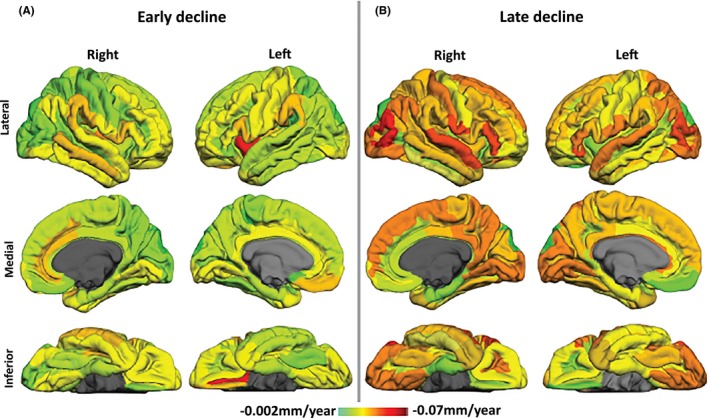
Illustrative rates of cortical thinning (mm/year) in the Early and Late Groups. In the Early stage group, the left insula (−0.072 mm/year) and medial olfactory sulcus (−0.063 mm/year) show high rates of atrophy, while the left orbitofrontal region (−0.022 mm/year) shows a slower rate of −0.022 mm/year. After the first decade, all cortical regions, especially the posterior regions, begin to atrophy much faster compared to the early stages

Cortical thinning rates (mm/year) for the Early and Late groups are depicted in Fig. 1, where the rate of cortical thinning ranged between −0.002 mm/year to −0.072 mm/year. While most cortical regions in the early stage had a rate of <−0.002 mm/year, the left insular short gyrus and the left olfactory sulcus decreased faster at a rate of −0.072 mm/year and −0.063 mm/year, respectively, while the left orbital gyrus decreased at a slower rate of −0.022 mm/year. In the Late‐stage group, greater rates of cortical thinning (>−0.002 mm/year) were evident in more widespread regions, but were especially higher in more posterior cortical regions. These include the cuneus (left: −0.038, right: −0.025), superior occipital cortex (left: −0.049, right: −0.038), middle occipital cortex (left: −0.049, right: −0.061), and superior parietal lobe (left: −0.038, right: −0.024). In addition, frontal regions such as the pars triangularis (left: −0.046, right: −0.039) and inferior orbital cortex (left: −0.06, right: −0.039) declined faster in later stage disease.

**Figure 2:**
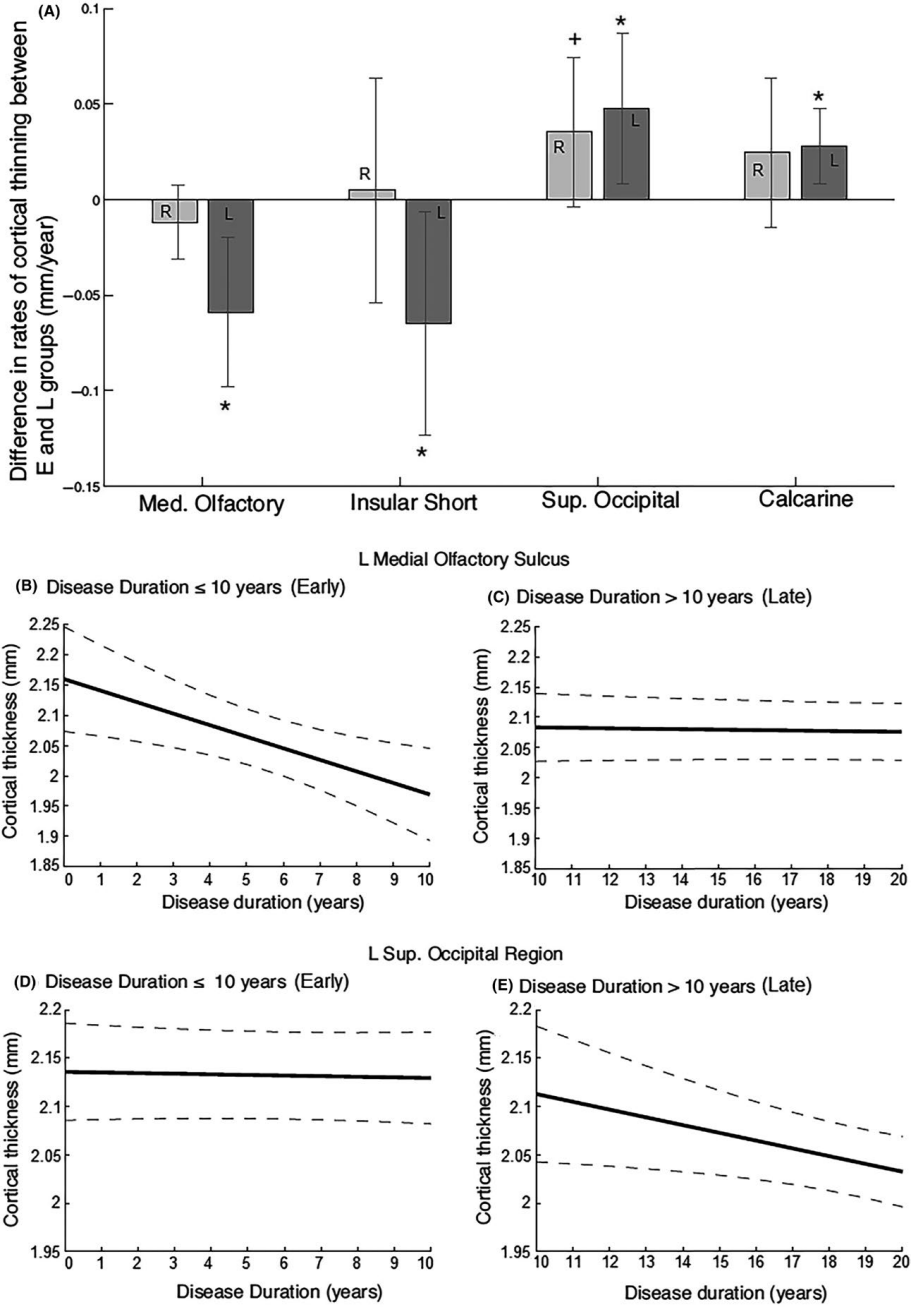
Regions with significant differences in the rates of atrophy between Early and Late stages of PD, [(rate of Late stage) – (rate of Early stage)]. In (A), right hemisphere regions are shown in light gray and left hemisphere regions are shown in dark gray. In (B–E), the solid lines represent the linear regression and the dashed lines represent the confidence intervals. (A) Rate differences of cortical thinning in the right (light gray) and left (dark gray) medial olfactory sulcus, insula, and the sup. occipital and calcarine cortex. Note that faster rates in early stages represent a negative value. (B, D) Cortical atrophy in the left medial olfactory sulcus is significantly faster in the first 10 years of PD. (C, E) Rates of atrophy in the left superior occipital region are more rapid after the first 10 years

Bootstrapped differences in rates of thinning between regions susceptible early versus later in PD showed significantly different rates of cortical thinning. These relationships are graphically represented in Fig. 2A. We have selected two anterior (left insular short gyrus and olfactory sulcus) and two posterior (left superior occipital and calcarine) regions to illustrate the different regional rates of decline in Early versus Late groups. Specifically, the rate of decline in the left insular short gyrus and the olfactory sulcus (Fig. 2B and C) is significantly higher in the first 10 years of PD, rather than in later years of disease. Similarly, cortical thickness in the posterior regions such as the left superior occipital (Fig. 2D and E) and calcarine regions decreases more rapidly and significantly after the first 10 years of PD. Remarkably, no such difference was noted in the right hemisphere at 95% confidence intervals.

### 3.4 Handedness and motor asymmetry

**Figure 3:**
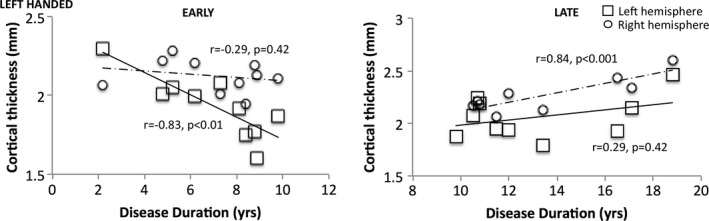
Cortical thickness in the medial olfactory sulcus in left‐handed patients. Early in the disease course, there is a significant correlation between disease duration and cortical thickness in the left hemisphere but not the right. The same relationship was found in right‐handed patients as well (Fig. S1)

To determine if cortical atrophy in the left hemisphere was consistent despite handedness or degree of motor asymmetry, we examined subsets of patients that differed based on handedness and side of motor predominance. First, we show that even in left‐handed patients, the rates of left hemisphere cortical atrophy, but not right hemisphere, are strongly correlated with disease duration in early duration patients (Fig. 3). This relationship is strongest in the left medial olfactory sulcus (*n *= 10 patients; *r* = −.83, *p *= .002). No such relationship exists in the right hemisphere. The same is true for right‐handed patients (see Fig. S1 for more details).

Secondly, we identified patients with motor asymmetry in the Off dopamine state. The laterality of motor symptoms was based on the difference between averaged UPDRS Off motor scores in the left and right extremities, where a negative score indicated predominant left‐sided symptoms. We included patients >1.0 S.D. from the mean of the total PD population; thus, identifying 32 patients with predominant right‐sided symptoms and 35 with left‐sided symptoms. These patients were evenly divided between early (*n *= 29) and late (*n *= 38) disease duration. This analysis confirms that the lateralized differences in Early stage patients are not driven by motor asymmetry. Despite the side of motor symptoms, cortical differences are negligible between hemispheres (see Table 3). Therefore, the greater left hemisphere changes in the Early stage cohort are not dependent on the lateralization of motor symptoms.

**Table 3:** Cortical thickness differences in the Insula and medial olfactory sulcus (mOlfS) in PD patients with marked lateralization of motor symptoms. Side of motor symptoms does not appear to influence the patterns of early left hemisphere degeneration, as cortical differences are negligible between hemispheres

Motor Lateralization	Left Insula (mm ± *SD*)^a^	Right Insula (mm ± *SD*)^a^	*p*‐value
Left side (*n *= 19)	3.38 ± 0.39	3.37 ± 0.32	.86
Right side (*n *= 10)	3.42 ± 0.34	3.28 ± 0.33	.04

	Left medial OlfS	Right medial OlfS	
Left side (*n *= 19)	2.09 ± 0.20	2.13 ± 0.16	.27
Right side (*n *= 10)	2.10 ± 0.28	2.18 ± 0.22	.05

a *SD*, standard deviation.

Overall, these results emphasize that there is a significant correlation between disease duration and cortical atrophy in left hemisphere regions early in the course of disease, which holds true regardless of handedness or side of motor severity. No such relationship was found for homologous regions in the right hemisphere.

## 4 Discussion

Cortical changes in PD appear to preferentially involve the left hemisphere early in the disease course. In the first decade of the disease, faster estimated rates of decline localize to the left insula, left olfactory sulcus, and to a lesser extent, the left orbitofrontal region. We note that this relationship is not dependent on handedness or influenced by the predominant side of motor symptoms. Our findings are in general agreement with other reports of left‐lateralized atrophy in PD (Bruck, 2004; Mak et al., 2014), and curiously mirror findings of left hemisphere predominance reported in other neurodegenerative disorders (Donix et al., 2013; Rohrer et al., 2012; Shi et al., 2009; Thompson et al., 2003; Whitwell et al., 2013). PD has long been recognized as an asymmetric disorder, with unilateral motor symptom onset as one of its cardinal features. Motor asymmetry in PD is associated with asymmetric nigrostriatal degeneration both in imaging and neuropathological studies (Kempster et al., 1989; Kumar, 2003; Wang et al., 2015) but the left nigrostriatal network appears uniquely susceptible to early degeneration (Prakash et al., 2012; Scherfler et al., 2012). The neuropathologic process in PD appears to act on different hemispheres and cortical regions, in different timescales.

The insula, olfactory cortex, and orbitofrontal region represent some of the earliest regions susceptible to alpha‐synuclein deposition according to the Braak model, and receive extensive synaptic inputs from monoaminergic projection neurons. The insula is emerging as an important hub of PD pathology, as it is uniquely susceptible to PD‐related neuropathology (Christopher et al., 2014), suffers volume loss early in PD (Ramirez‐Ruiz et al., 2005), and is associated with cognitive impairment related to attention and executive function (Mak et al., 2014). In particular, the insula receives dopaminergic projects from the mesocortical network originating in the midbrain ventral tegmental area and is thought to mediate cognitive flexibility (Gratwicke, Jahanshahi, & Foltynie, 2015). Degeneration of this network with loss of D2 receptors in the insula appears to correlate with executive dysfunction in PD (Christopher et al., 2014), and atrophy of the insular cortex has been associated with the development of dementia (Melzer et al., 2011). The insula is also a key region involved in central autonomic control (Beissner, Meissner, Bar, & Napadow, 2013), and its early degeneration in PD may thus contribute to autonomic dysfunction as well. Atrophy in the olfactory‐related cortex is also reported in patients within 10 years of disease onset (Lee et al., 2014), and reduced olfactory‐function is an established predictor of PD‐related pathology and symptoms (Jennings et al., 2014). Regional atrophy of the orbitofrontal cortex is also implicated in PD patients early in disease (Lyoo, Ryu, & Lee, 2010; Tinaz, Courtney, & Stern, 2011) and may explain the increased frequency of behavioral symptoms, such as anxiety and apathy, in early PD (Kostic & Filippi, 2011).

Why the left hemisphere exhibits such early and prominent susceptibility to atrophy remains an unanswered question; yet, other neurodegenerative disorders also show a similar pattern. In Alzheimer's disease (AD), the left hippocampus frequently shows earlier and more severe atrophy than the right (Donix et al., 2013; Shi et al., 2009; Thompson et al., 2003). Other left hemisphere regions including the entorhinal cortex, orbitofrontal, parietal regions, and periventricular regions are also vulnerable to atrophy early in AD (Janke et al., 2001), and 18‐F‐fluorodeoxyglucose positron emission tomography (PET) studies reveal greater temporoparietal cortical hypometabolism on the left (Loewenstein et al., 1989). Likewise, the left hemisphere is more susceptible to atrophy in behavioral and language variants of frontotemporal dementia (FTD). Left frontal lobe asymmetry is more notable in patients with behavioral variant FTD (Whitwell et al., 2013), and greater rates of left prefrontal and temporal atrophy are evident in progressive nonfluent aphasia and semantic dementia (Rohrer et al., 2012).

The etiology of this left hemisphere‐predominant atrophy across the spectrum of neurodegenerative disease remains unclear, although there are several hypotheses involving genetics, lateralized vulnerability, and disease‐specific factors. ApoE4 carriers, a well‐known risk factor for the development of AD, demonstrate regionally specific differences in left hemisphere brain morphology (Burggren et al., 2008; Liu et al., 2010). Left entorhinal cortex is smaller than the right in ApoE4 carriers as compared with noncarriers, regardless of age or cognitive status (Donix et al., 2013). This allele is associated with multiple pathologic processes that are thought to increase risk for AD (Mahley, Weisgraber, & Huang, 2006), and its asymmetric influence on brain development and cortical structure in the left hemisphere may confer an additional susceptibility to the neurodegenerative process in AD (Donix et al., 2013). Even genetic variants implicated in FTD show a similar pattern, where patients with MAPT mutations have greater left‐sided frontal atrophy, while those with the GRN mutation had greater right‐sided atrophy, and C9ORF72 carriers tended to show a symmetric pattern of atrophy (Whitwell et al., 2013).

A second hypothesis involves lateralized vulnerability related to left hemisphere dominance. It is well recognized that the left hemisphere is highly specialized for the control of skilled actions, including planning and implementation of complex movements, bimanual coordination, motor learning, and the critical processes of language (Serrien, Ivry, & Swinnen, 2006). While left hemispheric dominance is best established in right‐handed individuals, the majority of left‐handers exhibit a strong left lateralization for both language and motor control (Pujol, Deus, Losilla, & Capdevila, 1999; Rasmussen & Milner, 1977; Serrien & Sovijärvi‐Spapé, 2015). Furthermore, motor lateralization and handedness are associated with inherent asymmetries to the nigrostriatal dopamine system. A PET study in right‐handed Parkinson's patients showed that the degree of right hand preference correlates with dopamine levels in the left putamen, which subsequently relates to activation of the left SMA (de la Fuente‐Fernández, Kishore, Calne, Ruth, & Stoessl, 2000). Left lateralization in the nigrostriatal system is further corroborated by connectivity studies showing asymmetric coactivation of the left putamen with the ventrolateral and ventroanterior nuclei of the thalamus, areas that are critical for the control of voluntary movement (Postuma, 2005). This lateralization in the left cortico‐basal ganglia networks may provide some insight into their susceptibility to neurodegeneration in Parkinson's disease. Some have suggested that this could be a consequence of increased metabolic demand, resulting in increased oxidative stress and neurotoxicity (van der Hoorn et al., 2011). Another hypothesis is that increased excitatory corticostriatal projections may over time lead to excitotoxic effects and a greater dopaminergic deficit on the left as compared with the right (van der Hoorn et al., 2011).

However, it is important to note that not all of the differences we found in our cohort were left‐lateralized. Many right hemisphere regions also showed higher rates of atrophy as well, particularly later in the disease course. In particular, the anterior cingulate, frontal pole, inferior supramarginal gyrus, and the subparietal sulcus showed increased right‐lateralized atrophy in the Late‐stage group as compared with Early patients. Interestingly, these regions closely parallel those involved in the well‐described fronto‐parietal attentional control network (Wang et al., 2010). Neurodegeneration of this network may be responsible for the unique right‐lateralized cortical changes in PD. Similar findings of atrophy in these areas have been previously reported in PD patients (Madhyastha et al., 2015) and have been linked to cognitive impairment (Danti et al., 2015; Hwang et al., 2013; Mak et al., 2015). Our findings suggest a distinct susceptibility of bilateral occipital and right hemisphere degeneration that appears later in the disease course. Progressive cognitive symptoms in PD include visuospatial and attentional deficits, and this well‐described clinical finding may explain this cortical pattern. Alternately, this finding could suggest subtype differences within PD. In particular, since the early and late duration groups had similar motor severity, severe enough to be evaluated for DBS, patients with a “faster progression” of disease may have a more left‐lateralized pattern of degeneration, while those with longer progression may have widespread degenerative pattern, and subsequently reduced right‐sided and occipital thickness. Future longitudinal studies may help differentiate lateralized hemisphere changes to clinical symptomatology.

We acknowledge several limitations to our study. This was a cross‐sectional study, and therefore rates of atrophy are estimated. The technique we used for this is well‐validated and widely accepted. In fact, the majority of the studies assessing cortical thickness thus far, even those involving longitudinal measures, have actually been based on cross‐sectional analyses. This includes the original study published by Fischl (Fischl & Dale, 2000) as well as many others assessing cortical changes with age (Fjell et al., 2009), Alzheimer's disease (Fotenos, Snyder, Girton, Morris, & Buckner, 2005), and Parkinson's disease (Zarei et al., 2013). Few longitudinal studies of regional cortical thickness changes have been performed, and the data from these have generally been consistent with cross‐sectional estimates (Fotenos et al., 2005; Resnick, Pham, Kraut, Zonderman, & Davatzikos, 2003). Cross‐sectional designs have other limitations as well, including susceptibility to bias and the potential for confounding factors. However, there are also significant disadvantages to longitudinal studies, including subject drop out and the difficulty of collecting data over the years to decades needed to study neurodegenerative diseases. Furthermore, longitudinal neuroimaging studies present their own unique challenges, as changes in scanning and measurement technology over the study period can affect cortical thickness estimates and outcomes (Fjell et al., 2009; Han et al., 2006).

In conclusion, our findings lend support to a left hemisphere‐predominant pattern of cortical atrophy in PD, which is particularly evident early in the disease course, while more posterior and right hemisphere degeneration, later in the disease. While the underlying mechanisms are unclear, it appears that the unique specialization of the left hemispheric function appears to be somewhat of a double‐edged sword, giving rise to the extraordinary abilities of language and highly skilled movement, while also incurring an increased vulnerability to neurodegenerative processes. Further investigation is needed to better understand the underlying differences between cerebral hemispheres and increased susceptibility to neurodegenerative changes.

## Conflicts of Interest

The authors have no relevant conflicts of interest to report and no other relevant financial relationships to disclose other than listed.
