# Supplementary figures and images for: Cortical asymmetry in Parkinson's disease: early susceptibility of the left hemisphere
Source: Brain Behav. 2016 Oct 6;6(12):e00573. doi: 10.1002/brb3.573 (PMC5167000; doi:10.1002/brb3.573)

Supplemental Figure

B: RIGHT HANDED

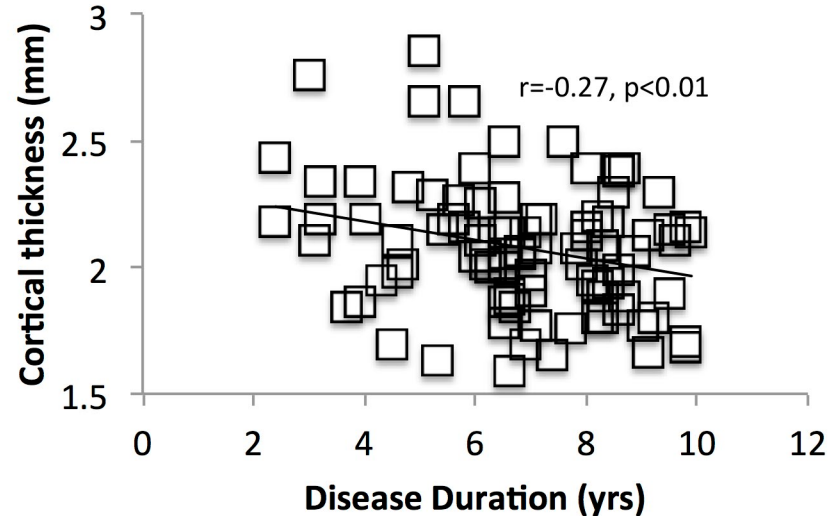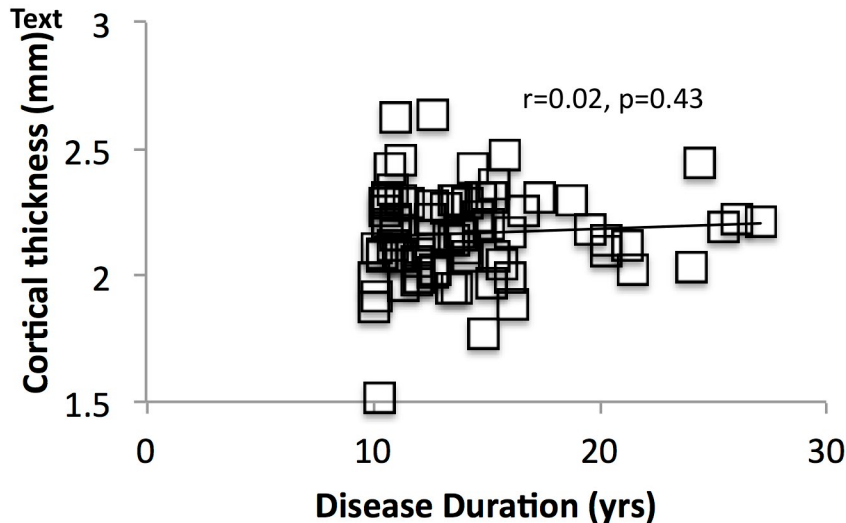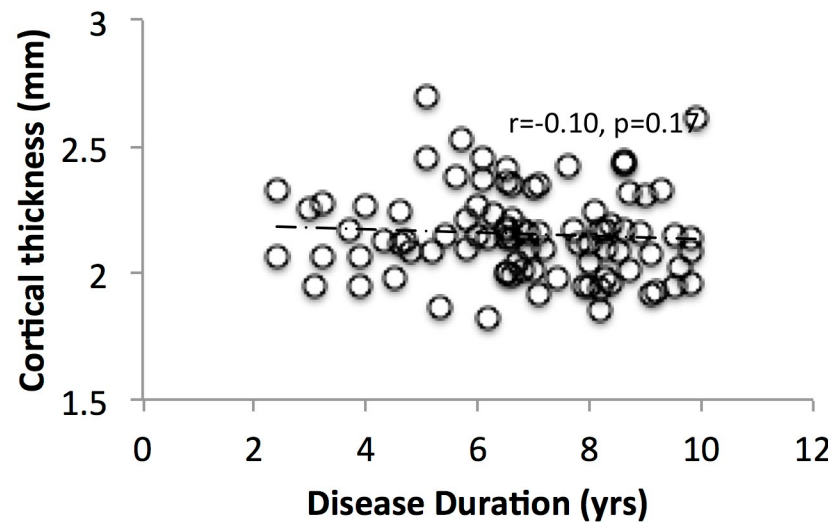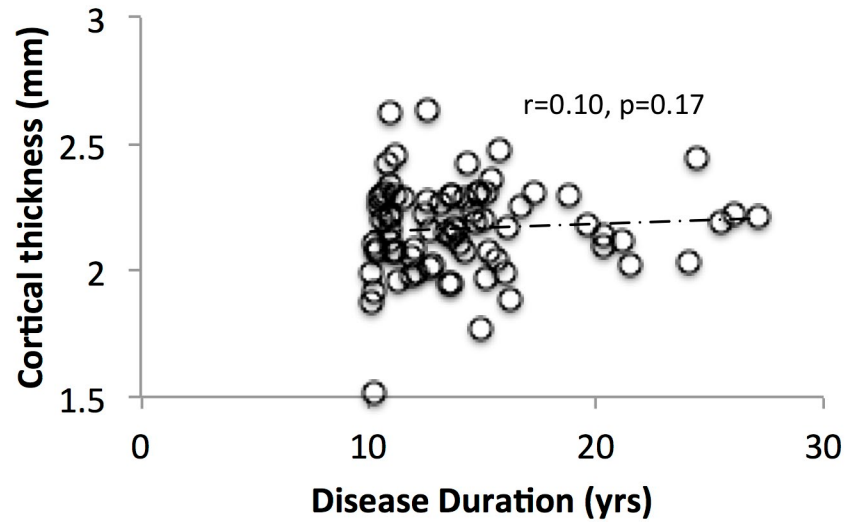

Supplement: Supplementary file 1 [file BRB3-6-e00573-s001.pdf]
